# Optimized Continuity of Care Report on Nursing Compliance and Review: A Retrospective Study

**DOI:** 10.3390/nursrep14030156

**Published:** 2024-08-23

**Authors:** David Luna-Aleixos, Lorena Francisco-Montesó, Marta López-Negre, Débora Blasco-Peris, Irene Llagostera-Reverter, María Jesús Valero-Chillerón, Ana Dolores Cervera-Pitarch, Andreu Gallego-Clemente, César Leal-Costa, Víctor M. González-Chordá

**Affiliations:** 1 eNursys Research Group (Code 162), Foundation for the Promotion of Health and Biomedical Research in the Valencian Region (FISABIO), 46020 Valencia, Spain; luna_davale@gva.es; 2 Hospital Universitario de La Plana, 12520 Vila-Real, Spain; francisco_lor@gva.es (L.F.-M.); lopez_marneg@gva.es (M.L.-N.); blasco_deb@gva.es (D.B.-P.); gallego_andcle@gva.es (A.G.-C.); 3 Joint Research Unit NURSIA (“NURSing Care, Information Systems, Tecnology and Quality”) FISABIO-UJI, 12071 Castellón de la Plana, Spain; vchorda@uji.es; 4 Nursing Research Group (GIENF Code 241), Nursing Department, Universitat Jaume I, 12071 Castellón de la Plana, Spain; 5 Centro de Salud de Burriana, Av. Nules, 31, 12530 Burriana, Spain; cervera_anapit@gva.es; 6 Faculty of Nursing, Campus de Ciencias de la Salud, University of Murcia, 30120 Murcia, Spain; cleal@um.es; 7 Nursing and Healthcare Research Unit (INVESTÉN-ISCIII), Institute of Health Carlos III, 28029 Madrid, Spain; 8 Network Biomedical Research Center on Frailty and Healthy Aging (CIBERFES), Institute of Health Carlos III, 28029 Madrid, Spain

**Keywords:** nursing, nursing records, continuity of care, healthcare quality

## Abstract

The Continuity of Care Report (CCR) is a fundamental document for ensuring high-quality healthcare and a smooth transition between different levels of care. The aim of this study was to evaluate the impact of optimizing the CCR to improve its completion rate by hospital nurses and its review by primary care nurses. To achieve this, a retrospective observational study was conducted on patients discharged from the University Hospital of La Plana de Vila-real during two three-month periods, one prior to the CCR improvement (2022) and one after (2023). No increase in the completion rate for the CCR was observed following its optimization (*p* = 0.226). However, a statistically significant improvement was noted in the percentage of reports reviewed (*p* > 0.001), increasing from 4.4% (*n* = 49) in 2022 to 30.5% (*n* = 327) in 2023. These results indicate that the optimization of the Continuity of Care Report enhances the communication between specialized care and primary care professionals.

## 1. Introduction

Among the challenges faced by healthcare professionals is providing high-quality healthcare [1]. The documentation recorded by nurses in their daily care activities provides important information about the care patients receive and constitutes a significant quality indicator of the care provided [2]. Moreover, maintaining high standards in nursing records is crucial to ensuring the continuity of effective care and optimizing its outcomes [3].

Regarding this continuity of care, the Continuity of Care Report (CCR) is posited as a crucial component to facilitate a smooth transition in patient care and to promote multidisciplinary coordination between various levels of care [4]. This report represents the document through which information related to the patient is transferred, encompassing both the nursing care provided up to that point [5] and the care that would be indicated following the patient’s discharge from the hospital [6].

This report improves communication among professionals and facilitates care planning to ensure continuity in healthcare delivery [7]. It also increases patient satisfaction with the care they receive, helping to alleviate the discomfort caused by a break in care after hospitalization [8]. It is estimated that patients who receive the CCR are 10% more likely to feel satisfied with their care compared to others [9], and this satisfaction is an important indicator of care quality [10].

Some of the benefits endorsed by the proper completion and traceability of the CCR include a reduction in healthcare costs, as improved communication among professionals fosters efficient resource management by avoiding duplicate consultations, inappropriate referrals, hospital readmissions, and overburdening the healthcare system [11,12]. However, nurses perceive clinical documentation as an administrative burden [13]. This perception is due to the amount of data and redundancy of elements they must record, despite the computerization of medical records [14]. This situation affects the quality of the records, leading to a reduction in adequate quality and quantity standards [2]. Additionally, the increased bureaucratic workload, which is estimated to take up 25% of nursing care time, limits the time available for direct patient care, covering barely one-third of the workday [15]. The importance of direct patient care lies in its association with reduced mortality, increased quality of care, and user satisfaction [16].

Given the influence of nurses’ work on patient outcomes, it is crucial to create optimal conditions in records that facilitate the continuity of care and generate positive effects for the user. To achieve these goals, nursing documentation systems must include information that is valid, reliable, and meets established standards.

These changes in information systems offer numerous advantages when digital technologies are employed to enhance patient information access and communication across different levels of care [17]. In fact, Health Information Exchanges (HIEs) and the interoperability of information systems have a recognized potential to improve healthcare delivery and reduce costs, while also contributing to a holistic approach to individual care and supporting the continuity of healthcare services [18].

Various studies have focused on the importance of nurses completing the CCR [6,19,20] and the communication at different levels of healthcare [5,11]. In response to the problems arising from inefficient communication that affect the continuity of care, nurses themselves propose solutions that could be addressed through HIE systems, aiming to improve the transfer of information at the time of patient discharge [21]. However, no proposal, regardless of how promising it may appear in theory, can be deemed valid without being subjected to an evaluation of its effectiveness. On the other hand, improvements in healthcare processes are not always implemented using a systematic methodology, based on research findings in clinical practice, which results in these processes becoming irregular, inconsistent, and ineffective [22].

Therefore, conducting such evaluations in records is essential due to their impact on the quality of care [23]. Additionally, research has been carried out to evaluate strategies for improving nursing records [24,25]. This type of study provides information that can help healthcare managers identify problems and define effective strategies to enhance the quality of nursing records [26].

This study aims to demonstrate the importance of improving nursing documentation systems by exploring the impact of optimizing the Continuity of Care Report (CCR), both in terms of completion by nurses in hospitalization units and its review by primary care nurses. The findings obtained will not only allow for an understanding of the effectiveness of the current implementation but also facilitate the identification of areas for improvement and the formulation of specific training and awareness initiatives. Moreover, evaluating the current impact of the CCR will contribute to establishing a solid foundation for future initiatives, aimed at optimizing the quality and efficiency of electronic records used by nurses.

## 2. Materials and Methods

### 2.1. Design and Setting

An observational, retrospective study based on registry data was conducted. This study was carried out in the Health Department of La Plana (Spain). This department is located in the southern part of the province of Castellon and covers a population ranging between 200,000 and 230,000 inhabitants.

### 2.2. Participants and Sample

The study population comprised all patients discharged from the various adult hospitalization units at the Hospital Universitario de La Plana in Vila-real (Spain). The inclusion criteria were being 18 years or older and having been discharged during the established periods.

Patients discharged from units with a discharge report protocol different from that of the other units were excluded from this study. Specifically, the units of Pulmonology/Neurology and Urology/Otolaryngology were excluded due to not having a protocol for completing the CCR, and Obstetrics/Pediatrics were excluded due to performing this process differently because of the nature of the patients they serve. Also excluded were cases resulting in death, transfers to the home care unit, and discharges that did not return the patient to their usual residence.

To calculate the sample size, a recent study by Llagostera et al. [25] was used as a reference. This study aimed to evaluate the impact of a strategy to promote the quantity and quality of nursing records, a goal similar to that of the present study. This work covered nursing evaluations conducted over two three-month periods in adult hospitalization units, one prior to the strategy being evaluated and one afterward. Additionally, Russell and McNeill [27] considered evaluating 200 electronic records sufficient to audit the implementation of a care planning system.

Based on these two articles, a three-month period in 2022 (from September to November) was established as the pre-optimization period for the CCR and another period with the same months in 2023 as the post-optimization period for the report. On the other hand, the clinical documentation unit of the hospital confirmed that the monthly average of patients at the hospital, who met the established inclusion criteria, exceeded 500 cases. This ensured that, within the selected periods, we could achieve a sufficient number of records for conducting the evaluation.

### 2.3. Description of the Sample Selection Process

During the two periods established by the inclusion criteria, there were a total of 6558 hospital discharges, with 50.14% (*n* = 3288) corresponding to 2022 and 49.86% (*n* = 3270) to 2023. Patients under 18 years old accounted for 8.58% (*n* = 282) in 2022 and 8.1% in 2023 (*n* = 265). Thus, the total sample meeting the inclusion criteria was 6011 hospital discharges, 50% (*n* = 3006) from the 2022 period and another 50% (*n* = 3005) from the 2023 period.

**Figure 1:**
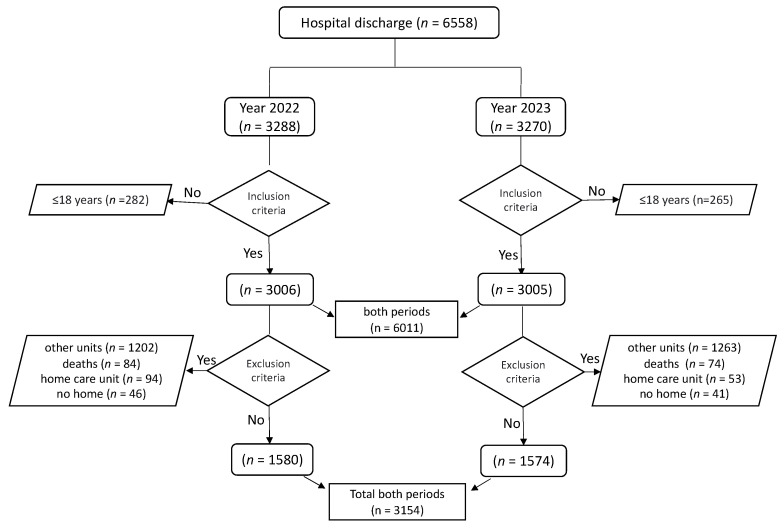
Sample selection process.

Of these discharges, 39.99% (*n* = 1202) in 2022 and 42.03% (*n* = 1263) in 2023 were excluded for belonging to hospitalization units not included in this study. There were 2.79% (*n* = 84) and 2.46% (*n* = 74) deaths in 2022 and 2023, respectively. Transfers to the home care unit accounted for 3.12% (*n* = 94) in 2022 and 1.76% (*n* = 53) in 2023. Lastly, 87 cases did not return to their home after hospital discharge, representing 1.53% (*n* = 46) in 2022 and 1.36% (*n* = 41) in 2023. After identifying all discharges that met the selection criteria, the final sample consisted of 3154 patients, with 1580 (50.1%) from the 2022 period and 1574 (49.9%) from the 2023 period (Figure 1).

### 2.4. Variables

This study included sociodemographic variables, such as sex and age; variables related to the care process, such as type of admission (emergency, scheduled), type of procedure (medical, surgical), and the hospitalization unit where the discharge occurred (Traumatology, Surgery/Gynecology, Cardiology/Digestive Medicine, Surgery, Internal Medicine); and variables related to the Continuity of Care Report (CCR), such as the existence of a CCR (yes/no), the creation date of the report (DD/MM/YY), the opening of the report by primary care nursing (yes/no), and the opening date of the report (DD/MM/YY).

#### Characteristics of the Optimization in the CCR

It is common for hospital clinical records and primary care records to be separate, increasing the risk of adverse effects after hospital discharge and making it difficult to provide high-quality healthcare [28]. This situation confirms the need to improve the transfer of information between different levels of care [29]. For this reason, it was decided to work on optimizing the Continuity of Care Report (CCR), which is the subject of analysis in this study, to achieve more efficient communication between hospital care and primary care professionals.

This optimization process was developed between January and May 2023. All the improvements implemented were in accordance with the guidelines stipulated in RD 1093/2010, of September 3, which approves the minimum dataset of clinical reports in the National Health System. One of the fields contemplated in the decree is based on adopting a nursing model to structure assessments. The model of the 14 basic needs of Virginia Henderson [30,31] was used as a reference, as it is one of the most widely used in hospitals in the Valencian Community [32].

With the aim of promoting greater implementation of the new CCR, a simpler and more parsimonious approach to completing the document was adopted [33], integrating it into the system used for creating clinical courses and enabling its completion, without the need for free-text entry. The final document presented an easy-to-read report, structured in a narrative format that clearly highlighted the patient’s needs and the prescribed care. To achieve this, predefined texts were developed based on the items that the nurse could select in the form. In this way, it became significantly easier to complete the CCR, saving time in the process and allowing the evaluation of each dimension required at the time of hospital discharge to be used as a checklist. Additionally, the new CCR automatically incorporates the results of the assessments conducted by nurses using the VALENF instrument [34], as well as information regarding injuries being treated during the hospital stay and the care of various devices that the patient needed to maintain after discharge. All this information was captured from the patient’s electronic health record (EHR), thereby preventing the loss of relevant information without the need to duplicate data entry.

Finally, to improve the accessibility of the document for primary care nurses, a system of alerts was established that automatically sent a notification to the nurse assigned to the patient at their health center upon completion of the CCR, providing direct access to the document generated by the hospitalization unit. This method of accessing the report allows primary care nurses to view the CCR without needing to leave their clinical management information system or access the program used in hospitalization. The greatest challenge of the improvement, which was nonetheless an essential requirement, was ensuring the interoperability of the CCR with the clinical information systems of the hospital units and the primary care systems. The new digital platform for completing the CCR can be filled out using the X-HIS^®^ software, version 4.12.1 (CSC, Northridge, CA, USA), which is the program used by the hospital nurses to manage electronic medical records, and it can be viewed through the ABUCASIS-SIA software, version 37.00.03 (AVS, Valencia, Spain), a system that comprehensively manages patient care in health centers under the Valencian Health Agency.

This work was made possible through the collaboration between the eNURSYS Research Group, the Nursing Research Group GIENF, and the health department’s IT service, which designed a custom digital platform for completing the CCR, stored in X-HIS^®^ according to the specifications provided by the nurses in the research group. Although this system cannot be commercialized or distributed to other hospitals with software different from that used at Hospital de La Plana in Vila-real, it represents an innovative approach to enhancing collaboration between IT departments in health services and clinical nurses in the development of digital platforms. These platforms are tailored to the software used by institutions and offer better outcomes compared to native interfaces.

### 2.5. Data Collection

The data were obtained from the hospital’s electronic medical records. A pseudonymized database was requested from the center’s IT department, excluding any personal data that could identify the patients. The structure of this database included all the variables under study and was agreed upon with the documentation service, which also retains the original database with patient identification.

### 2.6. Data Analysis Procedures

First, a descriptive analysis was conducted for each of the sample periods. Additionally, after verifying the applicability conditions of parametric tests, we studied whether there were significant differences between the two data collection periods, considering sociodemographic variables (age, sex), type of procedure (medical, surgical), and type of admission (scheduled, emergency). For the age variable, the Mann–Whitney U test was used. For the other variables, the chi-square test (χ^2^) was employed.

Second, in each period, the degree of completion of the Continuity of Care Reports was analyzed overall and for each hospitalization unit. Similarly, we explored whether there were statistically significant differences between the two periods regarding the access to the reports by primary care nurses. Both cases were analyzed using the chi-square test (χ^2^). A *p*-value < 0.05 was considered significant for hypothesis testing. The analysis was conducted using SPSS Statistics software, version 29.0.1.0 (IBM, Armonk, NY, USA).

### 2.7. Ethical Considerations

The project received approval from the center’s management and a favorable resolution from the Ethics and Research Committee in November 2023 (code: ICCENF. 30 November 2023). This study complies with Regulation (EU) 2016/679 of the European Parliament and of the Council of 27 April 2016, concerning the protection of natural persons, and Organic Law 3/2018 of December 5, on the Protection of Personal Data and Guarantee of Digital Rights. Specifically, it adheres to the second point of the seventeenth additional provision (section d), which considers the use of pseudonymized personal data for health research, particularly biomedical research, to be lawful.

To ensure the technical and functional separation between the research team and those responsible for pseudonymization, the hospital’s clinical documentation unit of was requested to provide a pseudonymized database, devoid of any personal data and containing only an identification code known solely to the documentation unit, not to the researchers. This was implemented to prevent potential re-identifications by third parties and to ensure the hospital’s ability to re-identify individuals in the event that a real and specific threat to the safety or health of a person or group of people was identified. Additionally, the researchers signed a responsibility agreement in which they explicitly committed not to engage in any re-identification activities of the study participants and to keep the database password-protected, refraining from sharing it through cloud services.

Based on this, the Ethics and Research Committee approved the request for exemption from informed consent.

## 3. Results

### 3.1. Descriptive Analysis of the Sample

Table 1 shows the descriptive analysis of the sample. The mean age of the cases included in this study was 66.17 (±17.77) years in 2022 and 65.25 (±18.44) years in 2023. The sample had a higher proportion of men (52.7% in 2022 and 50.4% in 2023), medical procedures (55% in 2022 and 57.2% in 2023), and emergency admissions (70.9% in 2022 and 71.6% in 2023). No statistically significant differences were observed between the two periods concerning sociodemographic variables and those related to the care process.

### 3.2. Completion of the Continuity of Care Report

Of the 3154 patients included in this study, 69.1% (*n* = 2179) had the Continuity of Care Report completed upon hospital discharge. Regarding the type of procedure, 68.5% (*n* = 1211) of medical procedures and 69.9% (*n* = 968) of surgical procedures had a report. According to the type of admission, 67% (*n* = 607) of scheduled admissions and 69.9% (*n* = 1572) of emergency admissions had the report completed at the end of the hospitalization process. Comparing the periods, there was a significant decrease in completed reports for surgical procedures (*p* = 0.003) and for scheduled admissions (*p* = 0.02), despite the nurses expressing that the new report was simpler and more efficient to complete.

Specifically, regarding the hospitalization units, in the 2022 period, the Traumatology (92.6%; *n* = 351) and Internal Medicine (89.1%; *n* = 122) units had the highest completion rates for the Continuity of Care Report. In 2023, the Traumatology (71.6%; *n* = 250) and Internal Medicine (79.9%; *n* = 175) units continued to have the highest completion rates. However, both units showed a statistically significant decrease, with the trauma unit exhibiting the most pronounced reduction (*p* <0.001). This finding supports the decrease in reports for surgical procedures and scheduled admissions, as these are typically the primary types of procedures and admissions handled by the trauma unit. The Surgery/Gynecology and Cardiology/Digestive units had the lowest completion rates in 2022 [53.7% (*n* = 195); 54.6% (*n* = 196)], but they experienced the most significant increases (*p* = 0.012; *p* < 0.001, respectively) in the 2023 period [63% (*n* = 218); 66.6% (*n* = 245)]. In this second period, the completion rates of the report showed greater homogeneity across all the services included in this study (Table 2).

### 3.3. Review of the Continuity of Care Report

Regarding the review of the reports by primary care nurses, significant differences were observed between the two periods, with respect to the hospitalization unit, type of procedure, and type of admission (*p* < 0.001) (Table 3). Specifically, in the 2022 period, 4.4% (*n* = 49) of the reports were reviewed by primary care nurses. This percentage increased to 30.5% (*n* = 327) in 2023. By hospitalization unit, all units showed an increase in the percentage of reports read by primary care, with the most notable being the Cardiology/Digestive unit, which went from 2% (*n* = 4) of reports reviewed in 2022 to 33.1% (*n* = 81) in 2023. Regarding the type of procedure, medical procedures saw the greatest increase, with 3.3% (*n* = 19) of reports reviewed in 2022, rising to 32.1% (*n* = 248) in 2023. Finally, for the type of admission, the increase in reviews of reports related to emergency admissions was noteworthy, rising from 3.7% (*n* = 29) in 2022 to 31.4% (*n* = 248) in 2023.

## 4. Discussion

The digitization of nursing records enables and expedites the evaluation of documentation used in care processes [35]. Similar to other studies that have assessed improvement strategies or the implementation of electronic records or care plans in hospitalization units [25,27], this study aimed to evaluate the impact, whether positive or negative, of the improvements introduced, in order to identify failures, barriers, or shortcomings that prevent nurses from reaching adequate quality standards. Unlike the aforementioned studies, this work did not observe an increase in the percentage of adoption of the new report; however, there was a statistically significant improvement in the review by primary care nurses, thus enhancing the communication channel between different levels of care.

Evaluating the recording systems used by nursing staff is crucial to identifying deficiencies and limitations that may hinder the effective provision of healthcare in general [36] and communication among professionals at different care levels. Therefore, healthcare institution managers must prioritize the evaluation of these systems [37] as a measure to detect errors and barriers in care procedures that could interfere, among other aspects, with evidence-based decision making [38]. Promoting a culture oriented toward total quality will allow for the application of quality at all stages of the process, regardless of the organizational level [39], with the aim of achieving the best health outcomes, preventing adverse effects, and improving healthcare delivery [40].

Regarding the completion of Continuity of Care Reports, the results did not show statistically significant differences between the two periods, despite one of the purposes of optimizing the Continuity of Care Report being to promote its completion for all patients discharged from the hospital. Saumalina et al. [41] indicated that motivation and supervision are determinants in the proper completion of nursing records. In this regard, it is noteworthy that, due to the arrival of a new management team at the hospital in September 2023, there were changes in the staff assigned to supervise different hospitalization units, including those whose completion rates significantly decreased, which may have been influenced by these changes. This reinforces the influence of supervision on the quality of electronic records, as noted by other authors [41,42,43]. Additionally, both units had high completion rates in 2022, suggesting that the demand to improve Continuity of Care Report completion rates, even when results are good, could demotivate nurses and lead them to place less importance on the proper completion of electronic records [44]. These changes also explain the decrease in report completion for patients from surgical processes and scheduled admissions, as this is a typical patient profile for the trauma unit, which experienced the largest decline. However, the greater uniformity in completion reflected in the results may be attributed to the simplicity and efficiency of completing the new CCR, in contrast to the previous platform used for the report, whose complexity could have posed a specific barrier in some of the hospital units.

Despite the importance of training for successfully implementing new procedures [45,46] and the type of communication used to gain acceptance from healthcare professionals [47], the optimization process did not consider any specific strategy that altered the traditional method of communicating changes in care procedures [48]. This reality, along with the resistance to change that healthcare professionals may exhibit [49], represents a significant barrier to the proper implementation of the Continuity of Care Report. However, caution should be exercised when determining the causes of these problems, and it would be advisable to conduct specific studies to evaluate the impact of these factors on the implementation of improvements in care procedures [50].

On the other hand, the results regarding the review of reports by primary care nurses showed statistically significant differences in all analyzed variables, with no other factors influencing this change, indicating that the optimization carried out to improve document accessibility provides evident benefits for the report to be used as an effective communication system between specialized and primary care. The data indicate that the previous report was a document that no one consulted and, therefore, did not achieve the goal of being a communication tool that ensured the continuity of care. The current report, supported by its IT system, may be an element that improves care continuity by providing quality information, thanks to its utility and the quality of the IT system that streamlines its completion and review [51]. Nevertheless, the percentage of CCR reviews after optimization is far from desirable, so it would be interesting to continue this line of research and address future studies on primary care nurses’ perceptions of the CCR, as well as exploring new optimizations to increase the review percentage.

However, all these findings raise questions about how objectives are evaluated in healthcare systems [52] and the importance of seeking appropriate indicators and analyses to effectively assess health systems. The current results are limited to measuring the quantity of actions performed, often without evaluating their utility and impact on patients. Finding suitable indicators would help detect the barriers and problems that hinder the provision of the best possible healthcare [53].

### Limitations

The present study has the limitation of being conducted in a single center, which may raise doubts about the possibility of extrapolating and comparing the results in other institutions. Similarly, the choice of the analyzed periods did not allow us to consider situations that could interfere with the results, such as changes in service supervisors or the study being conducted just after the end of the workers’ vacation period. Moreover, the sample selection may affect the generalization of the results due to the exclusion of two hospitalization units that did not complete the Continuity of Care Reports in the 2022 period.

However, the employed analyses could be adapted to evaluate various optimization processes in care procedures and, thus, be suitable for detecting problems and difficulties that hinder their proper implementation in healthcare institutions. Future research should analyze the impact of reviewing Continuity of Care Reports on the quality of healthcare and explore the influence of factors such as motivation and leadership on the successful implementation of improvements in care processes. It would also be appropriate to qualitatively analyze the perceptions of the nurses who complete and review the new CCR. In this way, ideas and areas for improvement could be identified that would help increase completion rates and enhance the report, ultimately improving the quality of healthcare provided.

## 5. Conclusions

Nurses can contribute significant improvements to the care processes in which they are involved; however, their effectiveness should be measured by appropriate indicators and not solely by completion rates or success metrics. The results indicate that optimizing the Continuity of Care Report improved communication between hospitalization and primary care nurses. Although the new version of the Continuity of Care Report is easier and more straightforward to complete, this improvement did not influence the percentage of patients for whom the report was completed at the time of hospital discharge. However, the old version was essentially a document within the electronic medical record that was rarely consulted and, therefore, of little use; whereas now, it facilitates interprofessional communication. These findings raise questions about the suitability of considering only the completion of the Continuity of Care Report as a quality indicator in the continuity of care, highlighting the need to adopt new methodologies to evaluate care processes. Future studies should examine the impact of the new CCR procedure on the quality of healthcare provided, as well as the perceptions of the nurses involved in completing and reviewing the report, and explore factors that influence the implementation of care processes.

## Figures and Tables

**Table 1:** Descriptive analysis of the sample.

Variable		2022	2023	
		m(ds) ^1^	m(ds) ^1^	*p*
Age		66.17 (17.77)	65.25 (18.44)	0.198 ^2^
		%(*n*) ^3^	%(*n*) ^3^	
Sex	Male	52.7 (832)	50.4 (793)	0.201 ^4^
	Female	47.3 (748)	49.6 (781)	
Process type	Medical	55.0 (869)	57.2 (900)	0.218 ^4^
	Surgical	45.0 (711)	42.8 (674)	
Admission type	Emergency	70.9 (1121)	71.6 (1127)	0.686 ^4^
	Scheduled	29.1 (459)	28.4 (447)	

^1^ Mean (standard deviation); ^2^ *p*-value: Mann–Whitney; ^3^ percentage (sample); ^4^ *p*-value: χ^2^.

**Table 2:** Completion of the CCR.

	2022		2023			TOTAL
Hospitalization Units	Total	CCR	Total	CCR		
	*n* ^1^	%(*n*) ^2^	*n* ^1^	%(*n*) ^2^	*p* ^3^	%(*n*) ^2^
Traumatology	379	92.6 (351)	349	71.6 (250)	<0.001	82.6 (601)
Surgery/Gynecology	363	53.7 (195)	346	63.0 (218)	0.012	58.3 (413)
Cardiology/Digestive	359	54.6 (196)	368	66.6 (245)	<0.001	60.7 (441)
Surgery	342	70.8 (242)	292	63.4 (185)	0.048	67.4 (427)
Internal Medicine	137	89.1 (122)	219	79.9 (175)	0.024	83.4 (297)
**Process type**						
Medical	869	67.2 (584)	900	69.7 (627)	0.265	68.5 (1211)
Surgical	711	73.4 (522)	674	66.2 (446)	0.003	69.9 (968)
**Admission type**						
Emergency	1121	69.8 (782)	1127	70.1 (790)	0.861	69.9 (1572)
Scheduled	459	70.6 (324)	447	63.3 (283)	0.02	67.0 (607)
**Total**	1580	70.0 (1106)	1574	68.2 (1073)	0.266	69.1 (2179)

^1^ *n*: sample; ^2^ percentage with CRR (sample); ^3^ *p*-value: χ^2^.

**Table 3:** Review of the CCR.

	2022		2023		
Hospitalization Units	Total	CCR	Total	CCR	
	*n* ^1^	%(*n*) ^2^	*n* ^1^	%(*n*) ^2^	*p* ^3^
Traumatology	351	6.6 (23)	250	30.4 (76)	<0.001
Surgery/Gynecology	195	4.6 (9)	218	29.8 (65)	<0.001
Cardiology/Digestive	196	2 (4)	245	33.1 (81)	<0.001
Surgery	242	4.1 (10)	185	29.7 (55)	<0.001
Internal Medicine	122	2.5 (3)	175	28.6 (50)	<0.001
**Process type**					
Medical	584	3.3 (19)	627	32.1 (201)	<0.001
Surgical	522	5.7 (30)	446	28.3 (126)	<0.001
**Admission type**					
Emergency	782	3.7 (29)	790	31.4 (248)	<0.001
Scheduled	324	6.2 (20)	283	27.9 (79)	<0.001
**Total**	1106	4.4 (49)	1073	30.5 (327)	<0.001

^1^ *n*: sample; ^2^ percentage of revised CRR (sample); ^3^ *p*-value: χ^2^.

## Data Availability

All necessary data are supplied and available in the manuscript; however, the corresponding author will provide the dataset upon request.

## Public Involvement Statement

There was no public patient participation.

## Guidelines and Standards Statement

This study was drafted in accordance with the STROBE guidelines for observational research.
